# Late‐Onset Diagnosis of Tuberous Sclerosis Complex Revealed by Renal Angiomyolipoma: A Case Report

**DOI:** 10.1002/ccr3.72928

**Published:** 2026-06-21

**Authors:** A. Fekih, W. Heni, C. Messaoudi, Y. Selmi, D. Touati, Z. Elloumi, J. Labidi

**Affiliations:** ^1^ Department of Nephrology Principal Military Hospital of Instruction of Tunis Tunis Tunisia

**Keywords:** adult‐onset, angiomyolipoma, giant angiomyolipoma, mTOR inhibitor, tuberous sclerosis complex, tumorectomy

## Abstract

Tuberous sclerosis complex (TSC) is a rare autosomal dominant disorder characterized by multisystem hamartomas caused by pathogenic variants in TSC1 or TSC2. It is typically diagnosed in childhood, most often because of neurological or dermatological manifestations. Diagnosis in late adulthood is uncommon and may pose significant diagnostic challenges. We report the case of a 59‐year‐old woman who presented with lumbar pain. Abdominal computed tomography (CT) revealed multiple bilateral renal angiomyolipomas (AMLs), more prominent in the right kidney. Further evaluation identified multiple facial angiofibromas and a periungual fibroma. Renal function was preserved, and no neurological, pulmonary, cardiac, or ophthalmological involvement was detected. Based on the International TSC Consensus diagnostic criteria, the diagnosis of TSC was established on clinical grounds. The patient underwent right renal tumorectomy without complications, and histopathology confirmed angiomyolipoma. Sirolimus therapy was initiated 3 months later to reduce the risk of progression of residual lesions. This case highlights that TSC may remain unrecognized until late adulthood and that renal angiomyolipoma can be the presenting feature. Clinicians should systematically evaluate adults with angiomyolipoma for underlying TSC, even in the absence of overt neurological signs. Early recognition is essential to ensure appropriate surveillance and prevent potentially serious renal and systemic complications.

## Key Clinical Message

A large renal angiomyolipoma in late adulthood may reveal previously undiagnosed tuberous sclerosis complex. Recognition of atypical age at presentation and significant tumor size should prompt thorough clinical and imaging evaluation to ensure timely diagnosis, multidisciplinary management, and prevention of serious complications.

**Abbreviations**
AML: angiomyolipoma
CT: computed tomography
LAM: lymphangioleiomyomatosis
mTOR: mammalian target of rapamycin
TOSCA: TuberOus SClerosis registry to increase disease Awareness
TSC: tuberous sclerosis complex
TSC1: tuberous sclerosis complex 1 gene
TSC2: tuberous sclerosis complex 2 gene

## 1 Introduction

Tuberous sclerosis complex (TSC) is a multisystem genetic disorder caused by mutations in the TSC1 or TSC2 genes, leading to dysregulation of the mammalian target of rapamycin (mTOR) signaling pathway and uncontrolled cellular proliferation [1, 2]. The disease is characterized by the formation of benign tumors affecting multiple organs, including the brain, skin, kidneys, heart, lungs, and eyes.

Although TSC is frequently diagnosed during childhood, some individuals remain undiagnosed until adulthood, particularly in cases with mild phenotypic expression. Recognition of extra‐renal features is essential for establishing the diagnosis according to the updated 2021 International TSC Consensus criteria [1].

Renal angiomyolipoma is one of the major features of TSC and can occasionally represent the revealing manifestation. The management of TSC is complex due to its multisystem involvement, characterized by significant neurological and developmental manifestations as well as potentially severe organ complications [3].

We report a case of bilateral renal angiomyolipomas leading to the diagnosis of previously unrecognized TSC in a 59‐year‐old woman.

## 2 Case History/Examination

A 59‐year‐old woman with a history of hypertension, asthma, and hypothyroidism presented with lumbar pain. There was no known family history of TSC.

**FIGURE 1:**
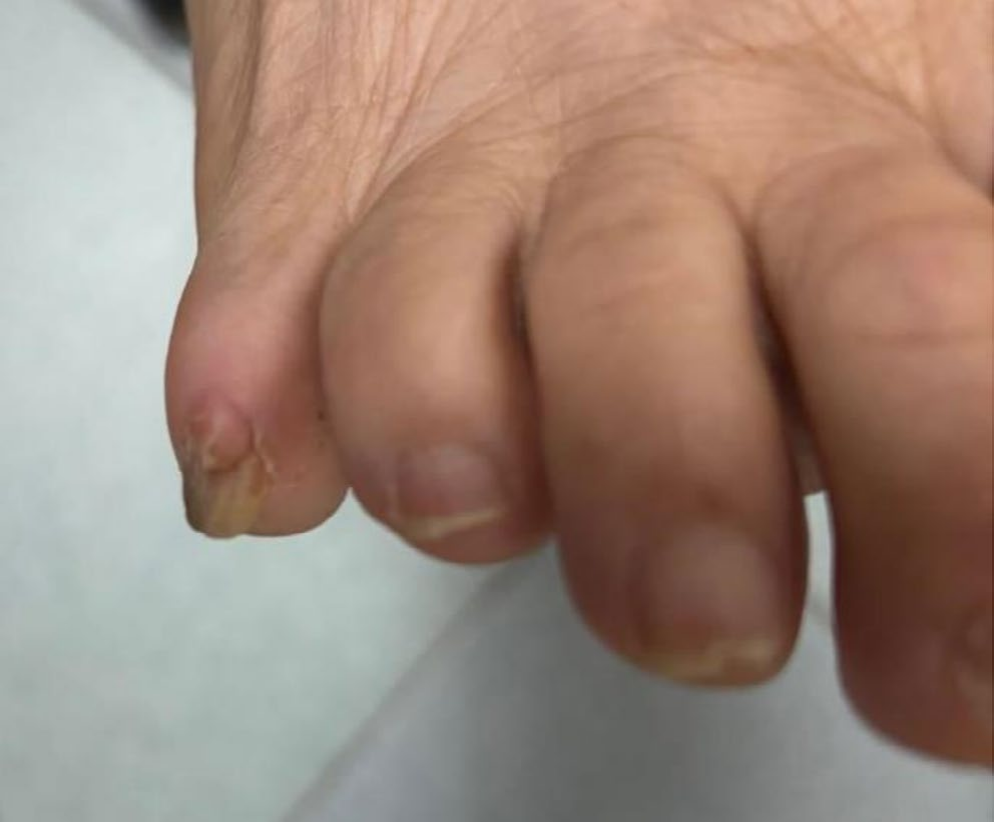
Periungual fibroma of the fifth toe in a patient with tuberous sclerosis complex.

On physical examination, the patient's blood pressure was 150/80 mmHg. She reported tenderness on right lumbar percussion. Dermatological evaluation revealed multiple facial angiofibromas, appearing as copper‐colored papules distributed over the malar regions, with ≥ 3 lesions. A periungual fibroma was observed on the left fifth toe (Figure 1). No hypomelanotic macules, shagreen patch, or other cutaneous stigmata were noted. The remainder of the systemic examination was unremarkable.

An initial abdominopelvic ultrasound was performed due to lumbar pain. It revealed multiple bilateral renal cortical nodules, more numerous and larger on the right side. These lesions were homogeneous, markedly hyperechoic, and showed no vascularity on Doppler imaging. The largest lesion measured 104 × 44 mm in the right kidney, while the largest left‐sided lesions measured 20 mm and 16 mm. Both kidneys were normal in size, with preserved corticomedullary differentiation and no evidence of hydronephrosis or nephrolithiasis.

## 3 Differential Diagnosis

The presence of multiple bilateral hyperechoic renal lesions on ultrasound initially raised the possibility of renal angiomyolipomas versus other renal neoplasms, particularly renal cell carcinoma, and prompted further cross‐sectional imaging for characterization. Given the association of multiple bilateral angiomyolipomas with hereditary syndromes, tuberous sclerosis complex (TSC) was suspected. The identification of multiple clinically typical facial angiofibromas and a periungual fibroma further supported this diagnosis. According to the 2021 International TSC Consensus diagnostic criteria, the diagnosis of TSC was established clinically based on the presence of two major criteria: multiple bilateral renal angiomyolipomas (≥ 2 lesions) and multiple facial angiofibromas [1]. Molecular analysis for TSC1 and TSC2 mutations could not be performed because genetic testing was not available at our center.

## 4 Investigations

Laboratory investigations showed preserved renal function, with a serum creatinine level of 56 μmol/L and blood urea of 4.9 mmol/L. Urinalysis revealed no hematuria or proteinuria. Serum electrolytes were within normal limits: sodium 138 mmol/L, potassium 4.3 mmol/L, and chloride 106 mmol/L. Serum calcium was 2.37 mmol/L and phosphate 1.12 mmol/L. Inflammatory markers showed a mildly elevated C‐reactive protein (CRP) at 9 mg/L. Total serum proteins were 70 g/L. Complete blood count revealed hemoglobin of 13.9 g/dL, white blood cell count of 6960/mm^3^, and platelet count of 310,000/mm^3^. Overall, laboratory findings indicated preserved renal function without proteinuria or hematological abnormalities (Table 1).

**TABLE 1:** Summary of laboratory and imaging findings at the time of diagnosis.

Parameter	Result
Laboratory findings	
Serum creatinine	56 μmol/L
Blood urea nitrogen	4.9 mmol/L
Sodium	138 mmol/L
Potassium	4.3 mmol/L
Chloride	106 mmol/L
Calcium	2.37 mmol/L
Phosphate	1.12 mmol/L
C‐reactive protein (CRP)	9 mg/L
Total serum proteins	70 g/L
Hemoglobin	13.9 g/dL
White blood cell count	6960/mm^3^
Platelet count	310,000/mm^3^
Urinalysis	No hematuria, no proteinuria
Imaging findings	
Largest lesion (right kidney—ultrasound)	104 × 44 mm
Largest lesions (left kidney—ultrasound)	20 mm and 16 mm
Right kidney size	12.8 cm
Left kidney size	11.7 cm

**FIGURE 2:**
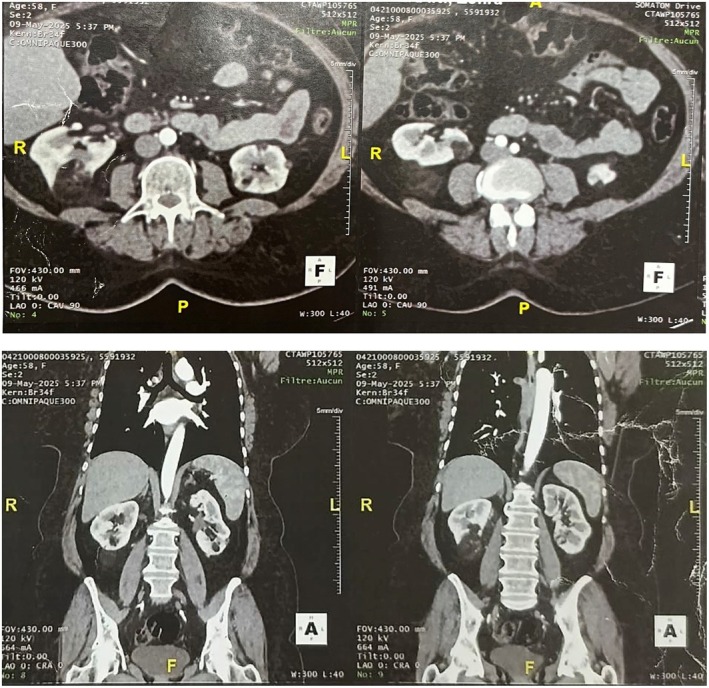
Contrast‐enhanced abdominal CT scan showing a large right renal angiomyolipoma with macroscopic fat and enhancing vascular components.

To further characterize the renal lesions and assess for systemic involvement, a contrast‐enhanced thoraco‐abdominopelvic and cerebral CT scan was performed. Abdominal CT confirmed the presence of multiple well‐circumscribed bilateral renal masses containing macroscopic fat with enhancing vascular components, consistent with angiomyolipomas, the largest measuring 54 × 33 mm axially and extending 97 mm craniocaudally in the right kidney. No radiological signs of hemorrhage or malignancy were observed (Figure 2).

Thoracic CT showed no pulmonary cysts or features suggestive of lymphangioleiomyomatosis. No mediastinal lymphadenopathy or pleural effusion was present. Brain CT demonstrated no cortical tubers, subependymal nodules, or other intracranial abnormalities. Overall, imaging supported bilateral renal angiomyolipomas without evidence of cerebral or thoracic involvement.

**FIGURE 3:**
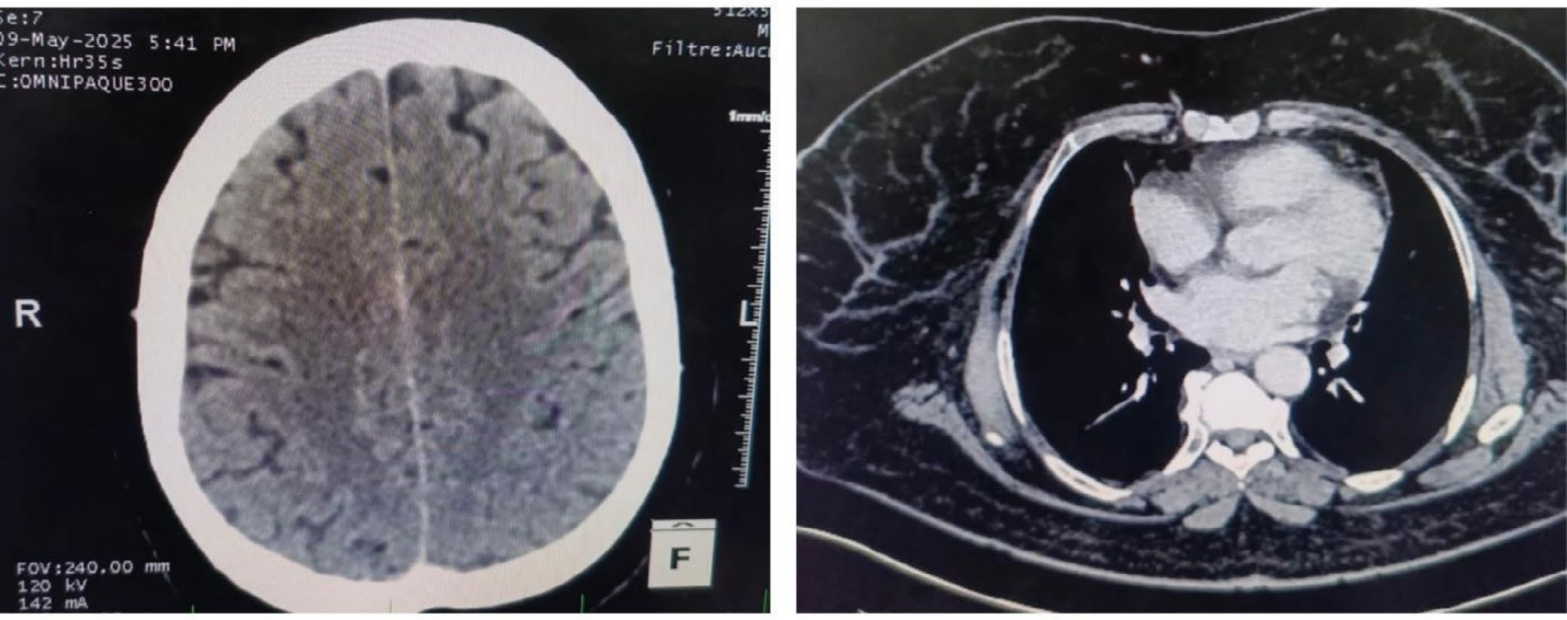
Representative thoracic and brain CT images demonstrating the absence of pulmonary cysts and intracranial lesions in this patient.

A comprehensive systemic evaluation was performed:

- Neurological assessment: A detailed neurological evaluation was conducted, including history for seizures, cognitive or behavioral changes, and a full neurological examination; brain CT was performed to exclude cortical tubers or subependymal nodules, and no abnormalities were detected (Figure 3).
- Cardiac evaluation: Transthoracic echocardiography revealed no cardiac rhabdomyomas.
- Pulmonary assessment: Thoracic CT showed no pulmonary cysts or features suggestive of lymphangioleiomyomatosis (LAM). Formal pulmonary function testing was not performed due to the absence of clinical symptoms (Figure 3).
- Ophthalmologic examination: Fundoscopic examination did not reveal retinal hamartomas.

## 5 Treatment

**FIGURE 4:**
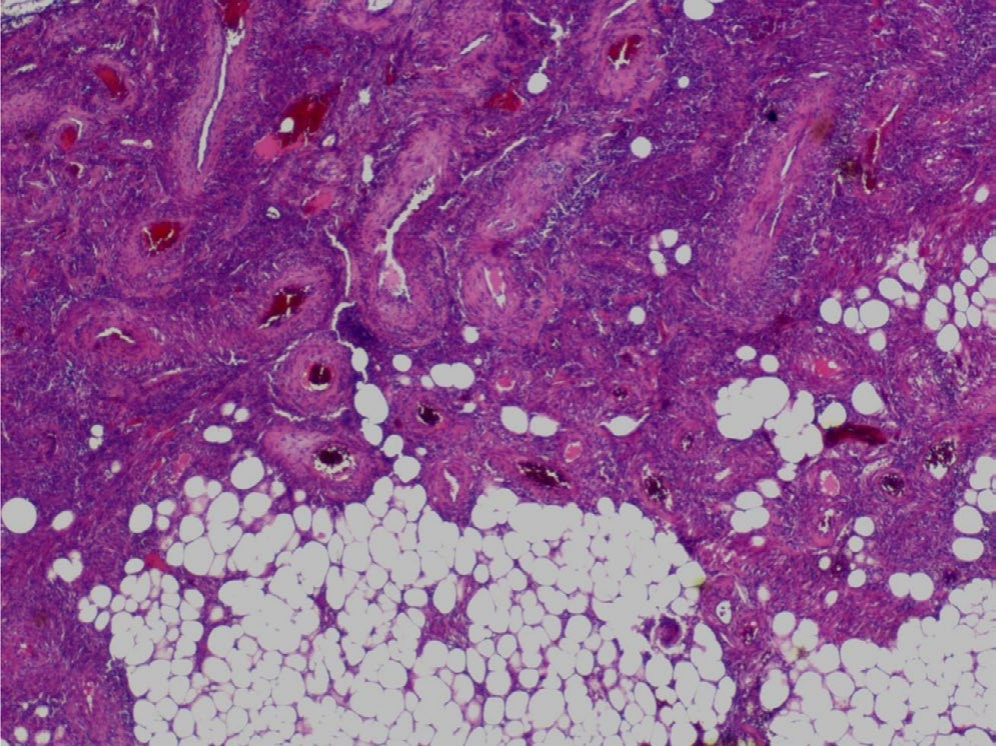
Histopathological examination of the renal angiomyolipoma showing the typical triphasic components (hematoxylin and eosin staining).

The right mass measured nearly 10 cm, well above the 4 cm threshold associated with a high risk of spontaneous hemorrhage. Although selective arterial embolization is commonly used for acute or moderately sized AMLs, imaging showed no active bleeding. A nephron‐sparing surgical approach (tumorectomy) was therefore selected to achieve definitive removal of this high‐risk lesion and to preserve renal parenchyma. Histopathological examination confirmed angiomyolipoma composed of adipose tissue, smooth muscle cells, and thick‐walled blood vessels, with no evidence of malignancy (Figure 4).

Postoperative laboratory assessments at 3 and 9 months demonstrated stable renal function (serum creatinine 65 μmol/L) and stable hematological and inflammatory markers. A control renal ultrasound demonstrated the persistence of smaller contralateral AMLs in the left kidney, without an increase in size compared to the preoperative evaluation. No lesions larger than 2 cm remained in the right kidney. These residual AMLs were considered at risk of progression, supporting the initiation of mTOR inhibitor therapy (sirolimus) 3 months after tumorectomy to reduce the risk of growth of residual lesions and to minimize future renal morbidity. The patient remains under multidisciplinary follow‐up.

## 6 Discussion

Tuberous sclerosis complex (TSC) is a multisystem genetic disorder characterized by highly variable clinical manifestations and a wide range of ages at diagnosis. In our patient, the diagnosis was made at 59 years old, which is notably later than the typical presentation. Most patients are diagnosed in childhood, particularly when neurological features such as seizures or developmental delays are present [1]. For example, in the international TOSCA registry, one of the largest international multicenter cohorts of patients with TSC, renal angiomyolipomas are most often diagnosed in childhood or adolescence, and the overall median age at initial TSC diagnosis was 1 year (range: 1–69 years) [4]. Similarly, other registry data indicate that renal angiomyolipomas are generally identified early in life, and that most patients develop renal manifestations long before reaching adulthood [5]. Although most TSC patients are diagnosed in childhood, rare cases diagnosed in adulthood have been reported, often with renal‐dominant phenotypes such as angiomyolipomas and subtle extra‐renal features [1, 6]. This pattern is consistent with the variable expressivity of TSC described in large cohorts and may be explained by mosaicism or milder phenotypic expression [7]. Renal angiomyolipomas (AMLs) are highly prevalent in women with TSC, even in the absence of lymphangioleiomyomatosis (LAM); epidemiological studies suggest that up to 80%–90% of adult women with TSC develop AMLs, whereas only a subset develop pulmonary LAM [4]. Documenting renal‐dominant cases without LAM, as in our patient, may help improve understanding of organ‐specific involvement and the pathogenesis of LAM, including the possible role of somatic mosaicism.

Physical examination in our patient revealed multiple facial angiofibromas and a periungual fibroma, which are among the clinical clues for TSC. The absence of hypomelanotic macules, shagreen patches, or other cutaneous stigmata underscores the variable expressivity of TSC. As previously reported, some patients may present with only a subset of the dermatological features, emphasizing the need for a thorough and systematic clinical evaluation in suspected cases [1, 4].

Renal angiomyolipomas represent the most common renal manifestation of TSC and may be the initial presentation in adults [8]. AMLs are benign mesenchymal tumors composed of varying proportions of vascular, smooth muscle, and adipose tissue components. Although typically benign, large lesions carry a risk of spontaneous hemorrhage [9].

Despite the large renal lesion in our case, no systemic involvement was identified. Brain imaging showed no cortical tubers or subependymal nodules, echocardiography excluded cardiac rhabdomyomas, thoracic imaging was negative for lymphangioleiomyomatosis, and fundoscopic examination was normal. This limited phenotypic expression highlights the organ‐specific and highly variable nature of TSC, even in older adults [1]. It also underscores the relevance of individualized management strategies based on the severity and location of organ involvement rather than age or genotype alone.

Management of renal AML depends on tumor size, symptoms, and risk of bleeding. While nephron‐sparing approaches and selective arterial embolization are preferred when feasible, surgical intervention may be required in selected cases. In our patient, the right AML measured 104 × 44 mm on ultrasound and 97 mm in height on CT, representing a large, high‐risk lesion. According to expert consensus and clinical guidelines, AMLs larger than 3–4 cm, those that are symptomatic, or those with a risk of hemorrhage warrant consideration for intervention due to increased risk of spontaneous bleeding and renal impairment [1, 3]. Although selective arterial embolization is generally recommended for large AMLs, the right renal mass in our patient measured nearly 10 cm, with substantial craniocaudal extension. Despite the absence of active bleeding, the lesion's size and vascular composition posed a high risk for spontaneous hemorrhage. Given preserved renal function and favorable tumor location, a nephron‐sparing tumorectomy was performed to definitively remove the dominant lesion, reduce recurrence risk, and preserve renal parenchyma, in accordance with current expert guidelines.

Following surgical management of the large right AML, systemic therapy with sirolimus, an mTOR inhibitor, was initiated. mTOR inhibitors are considered a cornerstone therapy for TSC‐associated renal angiomyolipomas, particularly for lesions ≥ 3 cm and/or with growth risk, those that are bilateral or multiple, and in patients for whom a nephron‐sparing approach is desirable [1, 3]. The mechanism of action of sirolimus involves inhibition of the mTORC1 pathway, which is dysregulated in TSC due to loss‐of‐function mutations in TSC1 or TSC2. This inhibition suppresses abnormal cell growth, angiogenesis, and the proliferation of AML cells. Clinical studies have demonstrated that sirolimus can reduce AML volume and stabilize renal function while maintaining a manageable safety profile [10], and similar efficacy has been demonstrated with everolimus in randomized and extension studies [11]. These findings support the use of mTOR inhibition as a key component of medical management in TSC patients with sizable or progressive AMLs.

In our patient, despite the absence of other systemic features of TSC, sirolimus was indicated to prevent growth of the contralateral smaller AMLs and to reduce the risk of future hemorrhagic or space‐occupying complications. This case illustrates the potential role of combining surgical intervention for large, symptomatic lesions with targeted mTOR inhibition in the management of adults with TSC, while acknowledging that long‐term postoperative data in this patient are not available.

Renal outcomes in adult TSC patients are highly variable and correlate with lesion burden, patient age, and the presence of bilateral or large renal angiomyolipomas (AMLs). In general, many adult patients with TSC maintain stable renal function, particularly when AMLs are small and asymptomatic; however, the risk of renal impairment increases with the size and number of AMLs and with recurrent bleeding events. Data from the TOSCA registry support that renal angiomyolipomas are among the most frequent and clinically significant manifestations of TSC, with renal morbidity increasing with age and lesion burden [4, 6].

## 7 Perspectives

This case supports the need for increased awareness of atypical or late‐diagnosed TSC forms and reinforces the importance of multidisciplinary management. Future perspectives include ongoing efforts to improve early and accurate diagnosis of TSC‐associated renal angiomyolipomas across all disease stages, as well as better identification and monitoring of other systemic manifestations of TSC. Such initiatives may facilitate timely intervention, risk stratification, and individualized management of affected patients.

## 8 Conclusions

Tuberous sclerosis complex (TSC) may remain unrecognized until late adulthood and can present with a giant renal angiomyolipoma. Early identification is crucial to ensure appropriate surveillance and to prevent renal complications, particularly hemorrhage. This case underscores the importance of individualized management for adults with TSC presenting with large renal AMLs. While surgical intervention and mTOR inhibition may be considered as part of the therapeutic strategy, further data are needed to evaluate long‐term outcomes in this patient population.

## Author Contributions

**A. Fekih:** conceptualization, data curation, formal analysis, methodology, writing – original draft, writing – review and editing. **W. Heni:** data curation, visualization. **C. Messaoudi:** data curation. **Y. Selmi:** data curation, visualization. **D. Touati:** validation, visualization. **Z. Elloumi:** data curation, visualization. **J. Labidi:** supervision, validation, writing – review and editing.

## Funding

The authors have nothing to report.

## Consent

Written informed consent was obtained from the patient for publication of this case report and accompanying images.

## Conflicts of Interest

The authors declare no conflicts of interest.

## Data Availability

The data supporting the findings of this case report are available from the corresponding author upon reasonable request, with appropriate consideration of patient confidentiality.
